# Whole-Genome Sequence Analysis of Pseudorabies Virus Clinical Isolates from Pigs in China between 2012 and 2017 in China

**DOI:** 10.3390/v13071322

**Published:** 2021-07-08

**Authors:** Ruiming Hu, Leyi Wang, Qingyun Liu, Lin Hua, Xi Huang, Yue Zhang, Jie Fan, Hongjian Chen, Wenbo Song, Wan Liang, Nengshui Ding, Zuohua Li, Zhen Ding, Xibiao Tang, Zhong Peng, Bin Wu

**Affiliations:** 1 State Key Laboratory of Agricultural Microbiology, The Cooperative Innovation Center for Sustainable Pig Production, College of Veterinary Medicine, Huazhong Agricultural University, Wuhan 430070, China; hrmvet19@163.com (R.H.); lqy948987886@163.com (Q.L.); hualin0@webmail.hzau.edu.cn (L.H.); hangxi-kiki@webmail.hzau.edu.cn (X.H.); zhangyue0309@webmail.hzau.edu.cn (Y.Z.); fanjred@163.com (J.F.); chenhongjian@webmail.hzau.edu.cn (H.C.); wbsong@webmail.hzau.edu.cn (W.S.); liangwan521521@163.com (W.L.); tangren77@126.com (X.T.); 2 Department of Veterinary Medicine, College of Animal Science and Technology, Jiangxi Agricultural University, Nanchang 330045, China; as188190731@126.com (N.D.); dingzhenhuz@jxau.edu.cn (Z.D.); 3 Jiangxi Provincial Key Laboratory for Animal Health, Jiangxi Agricultural University, Nanchang 330045, China; 4 Department of Veterinary Clinical Medicine and the Veterinary Diagnostic Laboratory, College of Veterinary Medicine, University of Illinois, Urbana, IL 61802, USA; leyiwang@illinois.edu; 5 Key Laboratory of Prevention and Control Agents for Animal Bacteriosis (Ministry of Agriculture), Animal Husbandry and Veterinary Institute, Hubei Academy of Agricultural Sciences, Wuhan 430070, China; 6 State Key Laboratory for Pig Genetic Improvement and Production Technology, Jiangxi Agricultural University, Nanchang 330045, China; 7 State Key Laboratory of Food Safety Technology for Meat Products, Xiamen 360000, China; 8 College of Veterinary Medicine, Hunan Agricultural University, Changsha 410128, China; ryj733@163.com

**Keywords:** pseudorabies virus, genome, phylogeny, recombination, selection pressure

## Abstract

Pseudorabies virus (PRV) is an economically significant swine infectious agent. A PRV outbreak took place in China in 2011 with novel virulent variants. Although the association of viral genomic variability with pathogenicity is not fully confirmed, the knowledge concerning PRV genomic diversity and evolution is still limited. Here, we sequenced 54 genomes of novel PRV variants isolated in China from 2012 to 2017. Phylogenetic analysis revealed that China strains and US/Europe strains were classified into two separate genotypes. PRV strains isolated from 2012 to 2017 in China are highly related to each other and genetically close to classic China strains such as Ea, Fa, and SC. RDP analysis revealed 23 recombination events within novel PRV variants, indicating that recombination contributes significantly to the viral evolution. The selection pressure analysis indicated that most ORFs were under evolutionary constraint, and 19 amino acid residue sites in 15 ORFs were identified under positive selection. Additionally, 37 unique mutations were identified in 19 ORFs, which distinguish the novel variants from classic strains. Overall, our study suggested that novel PRV variants might evolve from classical PRV strains through point mutation and recombination mechanisms.

## 1. Introduction

Pseudorabies virus (PRV), belonging to the family *Herpesviridae*, the subfamily *Alphaherpesvirinae*, is an economically important and devastating viral pathogen for the pig industry [1,2]. Pigs are natural and reservoir hosts of PRV [3,4]. PRV infection in pigs causes a wide range of clinical signs, including central neural disorder and high mortality in newborn piglets, neural disorder and respiratory diseases in nursery pigs, and abortion/stillbirth in sows [5]. It was documented that PRV infects a wide variety of hosts, except for higher primates and humans [3]. However, several recent studies reported that multiple human encephalomyelitis cases were linked to PRV in China, implying that PRV might be a zoonotic pathogen [6,7,8,9,10].

Because of the wide application of live-attenuated vaccines, PRV has been well controlled in most countries, including China [11,12,13]. Nevertheless, novel PRV variants with enhanced virulence broke out in pig farms in China in 2011 [14,15,16,17,18,19]. Animal experiments demonstrated that classic live vaccines failed to provide complete protection against novel PRV variants [10,12,14,17,20,21]. 

Comparative genome analysis revealed that virulent variant PRV acquired considerable mutations [14,15,16,18,22,23,24,25,26]. One study demonstrated that the genomic changes in gB contributed to the enhanced viral virulence of novel PRV variants [27]. However, the detailed genetic mechanisms of the enhanced virulence of PRV variants are still unclear. 

Intermolecular recombination is one of the major driving forces for herpesvirus evolution [28]. The genomic intermolecular recombination between two PRV strains has been reported in natural infection conditions by two studies [29,30]. Recently, several studies revealed the potential recombination between Bartha-K61 and virulent PRV strains, suggesting that recombination might have involved PRV evolution during the pandemic in China [31,32]. Additionally, recombination between virulent field viruses and the live vaccine strain was recorded in bovine herpesvirus 1 and infectious laryngotracheitis viruses [33,34]. These studies raised significant biosafety concerns about the application of live-attenuated vaccines. Most of these studies only identified recombination events in one strain. Whether recombination has been frequently involved in the evolution of other prevalent PRV strains is still unknown. 

Phylogenetic analysis showed that novel PRV variants are genetically close to China classic strains such as Ea, Fa, and SC. The mutations were found in various ORFs [15,22,23,24,25,35]. Furthermore, the phylogenetic and selection analysis of gB, gC, and gE revealed the significant enhancement of genetic diversity since 2011 and disclosed several adaptive mutation sites in gC and gB [36]. However, these studies mainly involved limited PRV genomes or only focused on several glycoproteins such as gB, gC, gE, and gD. The genomic sequence diversity of prevalent PRV strains and the selection pressure of most ORFs are still largely unknown. In summary, there are still several questions waiting to be addressed: (1) In recent years, the genome diversity of prevalent PRV strains in China is still unknown. Is there a new genotype that evolved during the PRV pandemic in China? (2) What is the genetic mechanism of PRV virulence enhancement? (3) What is the overall frequency of recombination in prevalent PRV strains? (4) The comprehensive profile of genetic diversity and selection pressure of each ORF of PRV is still unknown.

To address these questions, we employed the high-throughput sequencing (HTS) technique to sequence the genomes of 54 PRV strains isolated from 2011 to 2017. Together with 19 PRV genomes in GenBank, a total of 73 PRV genomes were included in the present study to analyze the PRV genomic diversity and evolution. At the full-genome level, we analyzed the genomic diversity, phylogenetic relationship, and the recombination of PRV strains from China, Europe, and the US. Additionally, we conducted ORF diversity and selection pressure analysis through all 67 ORFs. The selection pressure analysis of each ORF showed strong purifying selection pressure and identified multiple amino acid residues under positive selection. Furthermore, 37 distinct mutations, capable of differentiating novel PRV variants from classic strains, were identified in 19 ORFs, which would be useful for further genotype–phenotype studies.

## 2. Material and Methods

### 2.1. Viruses and Sequencing

Between 2012 and 2017, a total of 54 PRV strains were isolated from clinical samples of PRV-infected pigs in China. These samples were mainly abortive fetus or neonatal piglets with fatal neural disorders. These PRV strains came from 14 provinces of China. The geographical background of each PRV strain is listed in the Supplementary Materials (Table S1). All the PRV strains were isolated by PK-15 cells, and the passage times were strictly constrained within 5 times. Viral genome DNA was extracted as described previously [37]. Briefly, monolayers of PK-15 cells were infected with the PRV at an MOI of 10 and cultivated at 37 °C until a complete cytopathic effect was observed. Subsequently, the culture medium was collected without disrupting the cells and clarified by centrifugation at 4000× *g* for 10 min. Next, the viruses in the supernatant fluids were sedimented on a 30% sucrose cushion by ultracentrifugation at 77,000 × *g* for 2 h. The sedimented virion pellets were resuspended in sodium Tris-EDTA buffer. After the addition of proteinase-K (100 μg/mL final concentration) and sodium dodecyl sulfate (SDS; 0.5% final concentration), the lysate was incubated at 37 °C for 1 h, which was followed by phenol–chloroform extraction and ethanol precipitation. The white precipitation was viral genome DNA. The viral genome DNA was dissolved in TE buffer (pH = 8.0) and stored at −80 °C. Subsequently, the genome DNA of each PRV strain was subjected to DNA library preparation for high-throughput genomic sequencing (Illumina Hiseq 2500).

### 2.2. Assembly and Annotation of Genome Sequences

DNA library preparation was performed following the manual of the Nextera^TM^ DNA Flex Library Prep Kit (Illumina, CA). Libraries were sequenced on the HiSeq2500 (Illumina) platform. The paired-end sequencing gave a 150 bp reading length from each terminal. Full-genome consensus sequences were assembled using a pipeline reported previously, with some modifications [38,39]. Firstly, raw FastQ files were assembled de novo by using IDBA version 1.1.0 [40]. The contigs were further oriented and assembled by aligning the contigs against the genomic sequence of PRV reference strain NIA3 (KU900059.1), resulting in a draft genome sequence. The reads were mapped to this draft by using Maq [41], and the quality of the final assemblies was inspected by visualizing the alignment in Tablet [42] and manually corrected if necessary. At this stage, some genomes still showed a problematic assembly in the repetitive regions. To solve this problem, we used the iterative mapping approach [43]. Briefly, these regions were cut out from assemblies, and the separate contigs were extended and finally joined by iterative mapping of sequencing reads. The PRV genome contains two unique regions: unique long (UL) and unique short (US). The US region is flanked by large inverted and terminal repeat sequences (IR and TR). Considering that the length of each read (150 bp/reads) is much shorter than the inverted repeat sequences among the IR and TR regions, it is impossible to distinguish the reads from one copy within the IR region from the other identical copy located in the TR region. Hence, the sequencing data were assembled into a trimmed version of the genome, which only kept IR in the middle of the genome. These trimmed genome sequences were deposited in GenBank, and the accession numbers are listed in Table S1. The number of total clean reads, average sequencing depth, and coverage are summarized in Table S1. All the gapped regions are listed in the Supplementary Materials (Table S6). 

### 2.3. Multiple Sequence Alignment and Genome Annotation 

A multiple sequence alignment of 54 in-house-assembled viral genomes plus 19 genome sequences from GenBank (Table S2) was constructed by MAFFT version 7.221.3, option FFT-NS-I (maximum of 1000 cycles) [44]. Online PRV genomes used in this study are listed in Table S2. The TR regions of 19 reference genome sequences were also trimmed before all the analyses in the current study.

Genome annotation for 54 PRV genomic sequences was transferred from a genetically intact reference strain NIA3 (KU900059.1) by using RATT with a word size of 30, a cluster size of 400, a maximum extend cluster of 500, and an identity cutoff of 40 [45]. Annotation was manually inspected and corrected if the transfer was failed due to assembly gaps, sequence variability, or disruptive mutations. The annotated genomes of the newly sequenced PRV isolates were deposited in GenBank as partial genome sequences.

### 2.4. Genomic Diversity

**Figure 1:**
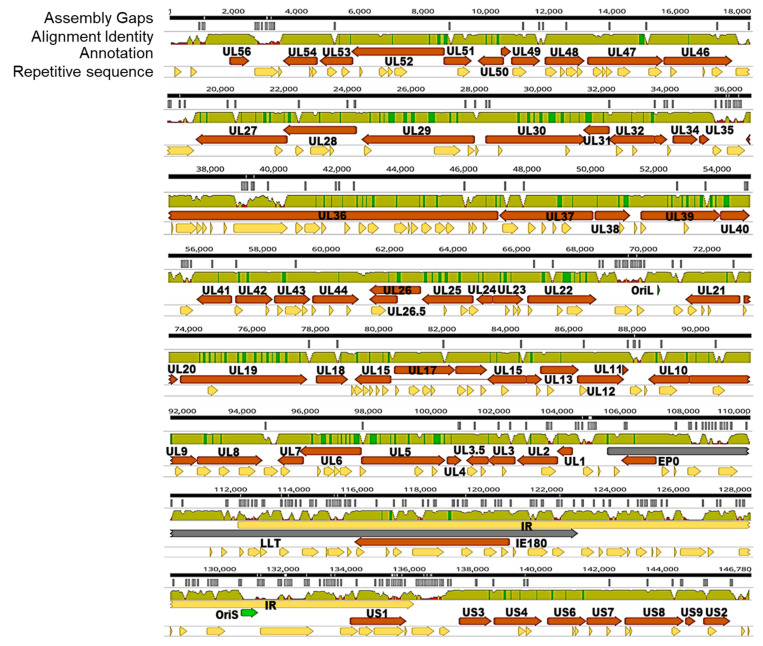
Overview of the sequence diversity in the alignment of 73 PRV genomic sequences. The alignment of 73 PRV genomic sequences was visualized by Geneious Prime. The black bar at the top represents a consensus sequence drawn from the alignment of 73 PRV genomic sequences. The white lines in the second row indicate the distribution of assembly gaps. In the third row, the curve of identity level is plotted from this alignment, which is colored as follows: green, 100% identity; green-brown, 30 to <100% identity; red, <30% identity. The fourth row is the annotation of the PRV genome. The bottom row shows the region of the repetitive sequence of the PRV genome.

Genome alignment was visualized by Geneious Prime 2020.02.1 (www.geneious.com, accessed on 5 March 2020), including genome annotation, alignment identity curve, gapped regions, and the regions of repetitive sequence (Figure 1). All the gapped regions were removed from alignment by Geneious Prime. The overall mean distance, indicating the arithmetic mean of all individual pairwise distances between each item in alignment, was measured by MEGA X (Jukes–Cantor model) with the pairwise deletion of gapped sites [46]. 

### 2.5. Phylogenetic and Recombination Analysis

A maximum likelihood (ML) phylogenetic tree was constructed by using MEGA X with the general time-reversible (GTR) model and 100 bootstrap replications [46]. Phylogenetic network analysis was performed using SplitsTree version 4.14.6 with the Jukes–Cantor model [47]. Recombination analysis was assessed by the Phi test in SplitsTree and further evaluated by the RDP 4 package [48]. All the PRV strains isolated in China plus Bartha-K61 were selected for RDP analysis. Considering that PRV was eradicated in domestic pig populations in most European and North American countries decades ago, classic Europe/US strains, except the live vaccine strain Bartha-K61, are highly impossible to show up in China pig farms. Therefore, we removed the Europe/US strains, except Bartha-K61, from the RDP recombination analysis. In the RDP analysis, 7 algorisms were considered, which are RDP, GENECONV, BootScan, Maxchi, Chimaera, Siscan, and 3seq. The recombination events were considered significant when at least 4 out of 7 algorisms showed *p* < 0.001. 

### 2.6. ORF Alignment and Divergence

Corresponding nucleotide or amino acid sequences of each ORF of every strain were extracted from the annotated genomes and pooled into one fasta file. Among the 67 ORFs, several ORFs contained gapped regions (Table S6). Those ORFs were excluded from the analysis. The sequences of each ORF were aligned at the codon level and the amino acid level by T-Coffee [49]. Nucleotide diversity was calculated by using MEGA X with all positions in alignment (Tamura–Nei model), and standard errors were calculated with a bootstrap procedure (100 replicates). Amino acid diversity was calculated by using MEGA X with the Poisson correction model [50].

### 2.7. Selection Pressure of Each ORF

In this analysis, nonsynonymous substitutions per nonsynonymous site (dN), synonymous substitutions per synonymous site (dS), and the dN/dS ratio were calculated by using the FUBAR algorism in the Datamonkey web server [51,52,53]. The individual site of each ORF under positive selection was evaluated by FUBAR [51], MEME [54], and CodeML of PAML [55]. Positive selection residues were at least confirmed by 2 out of 3 algorisms. The significance level of FUBAR is a posterior probability of >0.90. The significance level of MEME is *p* < 0.1. The significance level of CodeML is *p* < 0.05.

### 2.8. Data Availability

The 54 PRV genomic sequences in our study were submitted to GenBank, and the accession numbers are listed in Table S1. The accession numbers of the reference PRV sequences are listed in Table S2.

## 3. Result and Discussion

### 3.1. Genomic Sequencing, Assembly, and Alignment

To explore the genomic diversity of PRV-prevalent strains, 54 PRV strains were isolated from clinical samples from pig farms in China from 2012 to 2017. These samples were collected from 14 provinces in China. The viral genomic DNA samples of 54 PRV strains were subjected to high-throughput sequencing. The G+C% of PRV reference strain NIA3 was 74.0%, while the G+C% of 54 PRV strains ranged from 73.9% to 74.1%. The full genomic sequences of 54 PRV genomes and 19 online reference PRV genomes were aligned by MAFFT. Figure 1 shows a schematic diagram containing the PRV genome annotations, alignment identity curve, assembly gaps, and repetitive region. As shown in Figure 1, the distribution of nucleotide variation is uneven, and the highly variable regions usually are the regions containing repetitive sequences. Previous studies also showed a similar distribution in the genome diversity of HSV-1 and HCMV, indicating that the repetitive regions are highly variable [38,39,56]. 

### 3.2. Phylogenetic Analysis Indicates Strong Geographic Clustering

**Figure 2:**
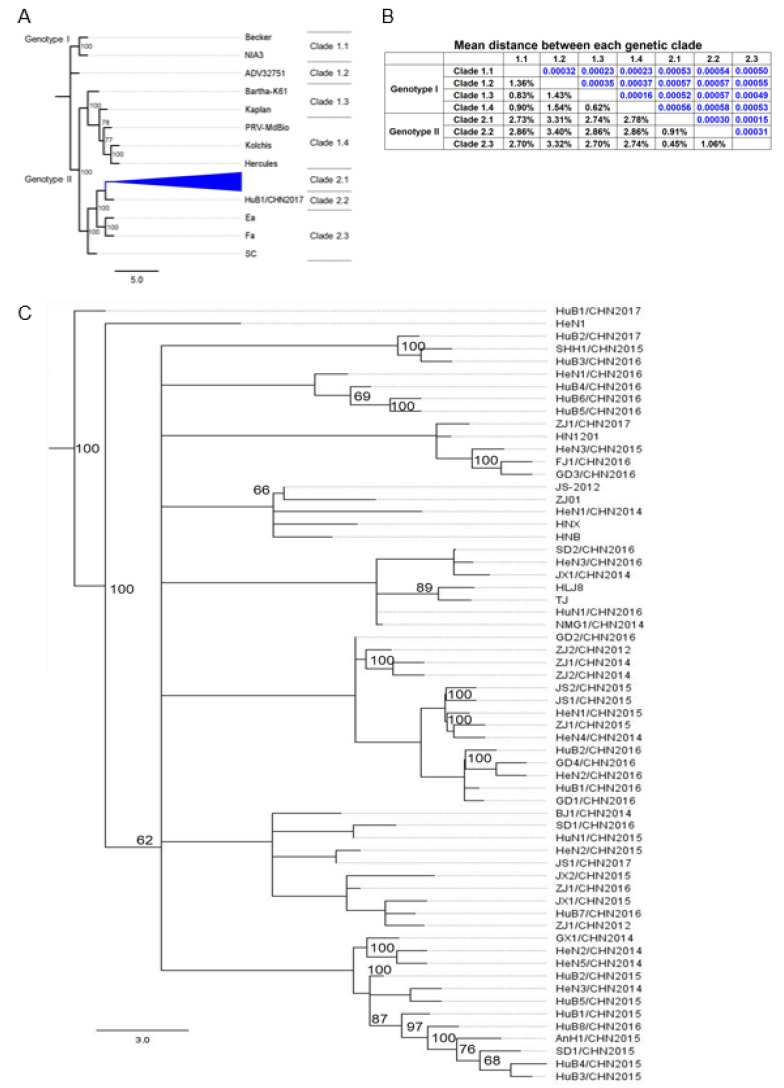
Phylogenetic relationship of PRV genome sequences. (**A**) A maximum likelihood (ML) tree was constructed by using MEGA X, based on the alignment of 73 PRV genome sequences (Tables S1 and S2). Gapped regions, which are labeled in Figure 1 within the multisequence alignment, were removed from all sequences before phylogenetic analysis. The ML tree is a rooted tree, and all branch lengths were drawn to a scale of nucleotide substitutions per site. Bootstrap resampling (100 replication) was performed. The branches of Genetic Clade 2.1 are collapsed. (**B**)The mean distance between each genetic clade was calculated by MEGA X with the Jukes–Cantor model. (**C**) Expansion of the collapsed clade (Clade 2.1) in the panel A of this figure.

In our study, phylogenetic analysis divided the PRV strains into two genotypes: Genotype I and Genotype II (Figure 2A). The Europe/US strains were clustered together forming Genotype I, while Chinese strains were all clustered as Genotype II, which is consistent with previous studies [14,24,25,35,57]. The mean genetic distance, representing the evolutionary distance of different genomes or genes from various strains, was measured by the number of nucleotide or amino acid substitutions between them. The overall mean distance of the whole alignment is 0.65%, and the genetic distance between Genotype I and Genotype II is 2.2%. The genetic distances between each genetic clade range from 0.36% to 2.6% (Figure 2B). Novel PRV strains prevalent in China are genetically close to local classic strains such as Ea and Fa, whereas newly sequenced Europe strains such as Herculus/Kolchis and ADV32751 are also close to Europe/US classic strains such as Becker and NIA3 (Figure 2A). The mean distance between two genotypes is substantially higher than the inner genotype mean distance (0.5–1%). This result is similar to previous findings, showing that the overall mean distances of PRV, BHV-1, EHV-1, EHV-4, and VZV are 1.65%, 0.81%, 0.79%, 0.16%, and 0.136%, respectively [57].

Within Genotype II, three strains (SC, Ea, and Fa) were isolated from the 1980s to 1990s in China [13]. As shown in Figure 2A, the strain SC formed a unique genetic clade (Clade 2.3), while classic strains Ea and Fa formed the major genetic clade (Clade 2.1) with other PRV strains isolated after 2011 in China, except strain HuB1/CHN2017. For a close observation, we collapsed Genetic Clade 2.1 in Figure 2A and displayed the phylogenetic tree of Clade 2.1 in Figure 2C. Interestingly, strain HuB1/CHN2017 formed a unique genetic clade (Clade 2.2), showing a substantial genetic distance against all other genetic clades in Genotype II (Figure 2B). 

Overall, these results indicate that the prevalent PRV strains in China are highly homologous, suggesting these strains might evolve from the same ancestor. However, the unique genetic clade was still detected in this study, which implied that PRV-prevalent strains continuously evolved during the PRV pandemic in China. The evolutionary mechanisms that drive novel genetic clades to emerge remain unknown.

### 3.3. Recombination between PRV Strains Is Robust

**Figure 3:**
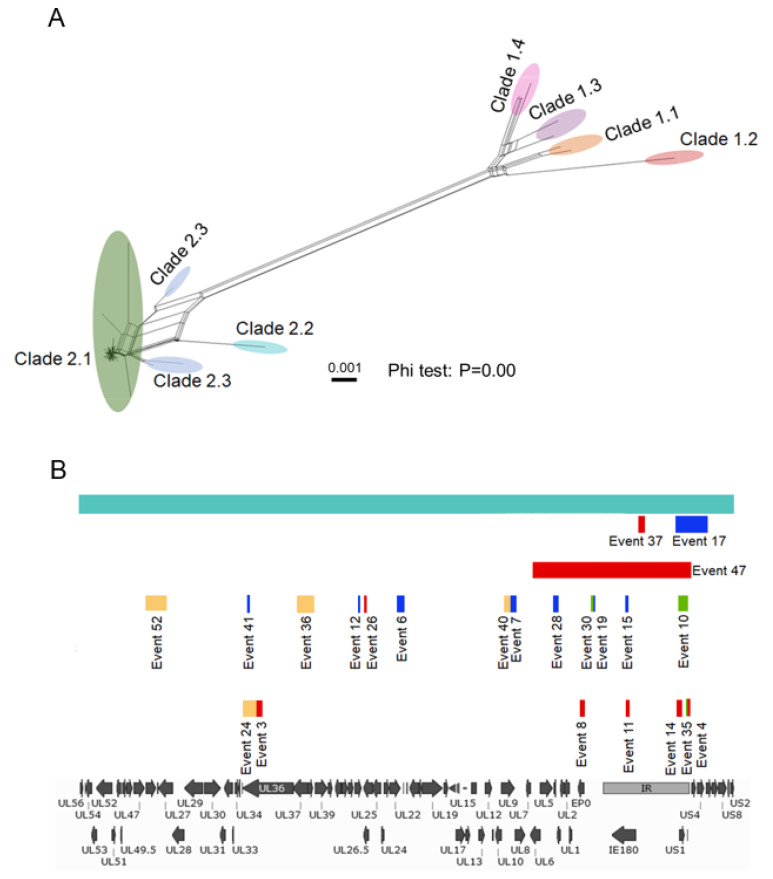
Recombination occurring pervasively in PRV newly sequenced strains. Recombination between PRV strains was determined by the Neighbor-Net split network and BootScan. (**A**) The Neighbor-Net split network was drawn by SplitsTree version 4.16 of 73 genomic sequences. The reticulate connections between each clade indicate events of recombination. Each clade was covered by a color scheme. The name of each strain is not displayed. (**B**) RDP analysis was applied to detect the recombination events in newly sequenced PRV genomes. All the recombination events are plotted in the schematic diagram. Each strip represents one recombination event and is labeled with one color. There are 4 colors in total, each representing different combinations of parental strains, as follows: red = Bartha-K61 + novel PRV variants; blue = Bartha-K61 + China classic strains, green = China classic strains + novel variants; yellow = novel variants + novel variants.

It has been well established that recombination contributes significantly to alphaherpesvirus evolution [57,58]. However, due to limited genome sequence availability, the profile of recombination within PRV-prevalent strains has not yet been clarified. In this study, we further investigated recombination between PRV strains by the SplitsTree and RDP package (Figure 3). The Phi test, which gives the statistical significance of the occurrence of recombination, strongly supported the existence of recombination (*P* = 0.00) (Figure 3A). Phylogenetic network analysis showed that recombination events between each genetic clade within Genotype II were more robust than Genotype I (Figure 3A). Recombination between two genotypes was also detected (Figure 3A). 

To further determine the distribution of recombination events within the newly sequenced strains, all the PRV strains isolated in China plus Bartha-K61 were selected for RDP analysis. All the recombination events are exhibited in Figure 3B, and detailed information concerning the location of recombination events, recombination parental strains, and the significance of each recombination event is listed in Tables S3–S5. In total, 23 recombination events were identified, of which only four events were recombination between two newly sequenced PRV strains (Events 24, 36, 40, and 52), whereas the others were recombination between two classic strains or recombination between a classic strain and a newly sequenced strain (Table S3). Additionally, the distribution of recombination events was not even. Clearly, the UL and US regions showed fewer recombination events, while the EP0 to IR regions contained significantly more recombination events (Figure 3B). One previous study also showed similarly that the IR and TR regions are recombination hot spots in HSV-1 [59].

It is worth noticing that Recombination Event 37, located in the IR region, was identified in 59 strains, while Recombination Event 17, spanning from the IR to US regions, was detected in 13 strains (Table S4). Other recombination events were only recognized in one strain (Table S3). Most recombination events occurred within the fragments shorter than 6 kb (Figure 3B); however, Event 47 detected in HuB1/CHN2017 showed a notably long recombination region starting from UL6 to the entire IR region (33kb), implying a different recombination mechanism in the evolutionary process of the novel genetic clade (Figure 3B).

Among all 23 recombination events, Bartha-K61 was involved in 16 events, including Event 17 and Event 37 (Table S3). The analysis results that showed the recombination between wild PRV strains and Bartha-K61 is possible, and more direct field evidence is ideally needed to support these results. Previous studies have demonstrated the high possibility of recombination between vaccine and clinically prevalent strains in different herpesviruses such as pseudorabies virus, varicella-zoster virus, bovine herpesvirus 1, infectious laryngotracheitis virus, and Marek’s disease virus [31,32,60,61]. Overall, these results indicate that recombination contributes to the evolution of novel PRV variants. It should be noted that recombination analysis based on software such as SplitsTree and RDP can only predict the potential recombination events among different viral strains and estimate the potential contribution of recombination during viral evolution in certain periods. To demonstrate the existence and the contribution of certain recombination events, more direct evidence such as detecting both recombination parental strains from the same animal is necessary.

### 3.4. Diversity of Protein-Coding Sequences

A smaller number of genomic sequences available limits the analysis of amino acid diversity of most ORFs. Previous studies only focused on several ORFs, such as UL44 (gC) and US6 (gD) [22,36,62]. In this study, we assessed the global diversity of PRV protein-coding sequences, including all 67 ORFs. 

As shown in Table 1, most ORFs are conservative, while only 11 ORFs show over 3% diversity. The relatively more conservative ORFs are UL30, UL19, UL22, UL5, and UL7, with diversity below 0.5% (Table 1). Within these proteins, UL30 and UL5 are key proteins of viral genome DNA synthesis [4]. UL19, also named VP5, is the major capsid component [4,62]. It is not surprising that those proteins are highly conservative, as they play critical roles during viral lytic replication. UL7 shows the lowest diversity. Although the exact function of UL7 remains unclear, it has been demonstrated that UL7 is involved in viral egress and virion release from the cytoplasmic membrane [63]. Viral glycoproteins are the major targets of neutralizing antibodies. Consequently, viruses with mutations in glycoproteins could evade host-neutralizing antibodies. In our results, only gE and gI exhibit a relatively high level of divergence, while other glycoproteins show relatively conservative. Nevertheless, care should be taken when comparing the diversity level of different ORFs, because the diversity at certain amino acid residues may truly reflect the functional diversity or epitope diversity, while the diversity at certain regions may not affect the neutralization epitope or protein function at all.

**Figure 4:**
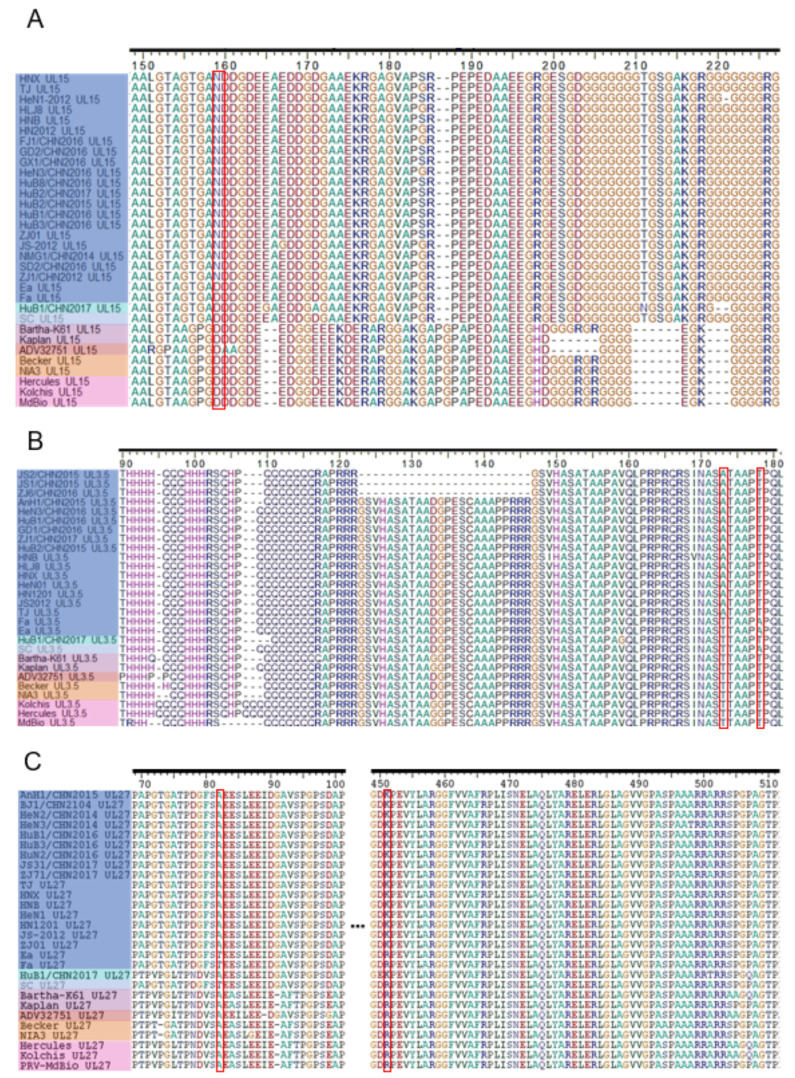
Presentative amino acid (AA) variation in UL15, UL3.5, and UL27. Partial regions of the amino acid (AA) alignment of UL15 (**A**), UL3.5 (**B**), and UL27 (**C**) are displayed in the three panels. To make the alignment easy to display, the redundancy of newly sequenced ORFs is reduced, while all the reference sequences are maintained. The panel of the strain name is covered by colored boxes, which represent their genetic clades with the same color scheme used in Figure 3A. The distinguishing mutations that distinguish PRV strains from classic strains are highlighted by red boxes.

The alignments in UL27, UL15, and UL3.5 chosen as their corresponding ORFs exhibit the variations seen in most PRV proteins in Table 1, including single amino acid variations, short insertion/deletion, and simple sequence repeat (SSR)-based variations. To easily view variations in the alignment, we removed the identical sequences among newly sequenced ORFs in this study (Figure 4). In the alignment of UL15, there are multiple mutations that distinguish all PRV isolates into Genotype I and Genotype II, such as GTGA155-158AGPG, AEDD165-169ΔΔDG, R185A, and RG198HD (Figure 4A). There are also multiple mutations representing the unique genetic background of HuB1/CHN2017 such as E165G, D171A, and T211N. Although most residues show more conservative in each genotype, aa184 shows variation within Genetic Clade 2.1 of Genotype II (Figure 4A). Only one unique mutation at aa159 was identified in UL15, changing from aspartate in classic strains to asparagine in novel PRV variants (Figure 4A).

In the UL3.5 alignment, the pattern of mutation was different. The major mutations occurred within the SSR region (aa90-146) (Figure 4B). Particularly, two distinct mutations were identified at aa 173 and aa 178 (Figure 4B). Interestingly, ZJ1/CHN2016, JS1/CHN2015, and JS2/CHN2015 showed short deletion at aa123-146 (Figure 4B).

In the alignment of UL27, Genotype I showed different amino acid sequences in Region aa70-91 from Genotype II, while a distinguishing mutation was located at aa82 (Figure 4C). Interestingly, UL27 of HuB1/CHN2017 showed a different amino acid sequence in this region compared with other strains in Genotype II. One study showed that replacing the UL27 of the PRV JS-2012 strain with Bartha-K61 caused the attenuation of viral virulence [27]. Therefore, we speculated that the variations in UL27 might contribute to both antigenic drift and virulence variation. This hypothesis is worth further investigation in the future.

Several studies have demonstrated that, compared with classic strains isolated decades ago, novel PRV variants possess enhanced virulence, including a larger plaque size, severer clinical signs, and higher mortality in pigs [14,16,24,35]. Although the correlation of viral genome variability with pathogenicity enhancement is highly possible, the exact causative mutation sites are hard to locate in herpesvirus due to limited genome sequences and many single mutations in a large genome. We speculated that the causative mutations leading to virulence enhancement in variant PRV strains might be included in the unique mutations, which are collectively different from all other classic strains. Therefore, identifying unique mutations is crucial to understanding viral evolution.

In the present study, we determined the unique mutations that distinguish novel PRV variants in China except HuB1/CHN2017 from classic strains in China (Ea, Fa, SC) or classic strains isolated in both China and Europe/US. Surprisingly, these unique mutations are sparse and sporadic (Table 2). Among the listed proteins, UL5, UL15, and UL42 are the key components for viral genomic DNA replication and packages. UL36, UL46, and UL47 are important tegument proteins, essential for virion assembly and egress. UL27, UL44, UL49.5, and US4 are envelope glycoproteins [4]. UL13 is a serine/threonine protein kinase that promotes viral egress and neural virulence [4]. UL27 (gB) is the receptor-binding protein, containing the most important neutralizing epitopes and strongly associated with PRV virulence [4,27]. Previous studies have identified several neutralizing epitopes of gB, such as aa59-126 and aa204-223 [64,65]. In our study, four distinguishing mutations (T82A, R451K, H560Q, T737A, V895A) were identified in gB (Table 2), of which was T82A located within epitope aa59-126, which may cause antigenic drift. 

UL36 is one of the major scaffold proteins in the tegument, which is also essential for viral replication and neural invasiveness [4]. It was shown that the most functional essential regions of UL36 are located at the N terminal, while aa 2087-2795 deletion only has a marginal effect on viral replication in vitro [66]. The only deletion region at aa6-225 caused a lower viral titer in PK15, while the PRV-virus-bearing deletion at aa226-299, aa2026-2970, and aa3055-3078 showed no viral replication in vitro [67]. As shown in Table 2, four distinguishing mutations (P/S292A, P/S/Q293A, A/G2068R, A3059/G), identified in UL36, are located in the indispensable region, while R2286Q is located in the dispensable region. Whether these mutations contribute to viral virulence enhancement is still unclear. Until now, the functional essential motifs or aa residues of most PRV ORFs are still unknown. Although it is unable to link the evolutionary diversity sites with biological functions in most ORFs, the ORF diversity profile identified in our study has shed the light on genotype–phenotype studies in the future.

### 3.5. Selection Pressure Act on Each ORF

**Figure 5:**
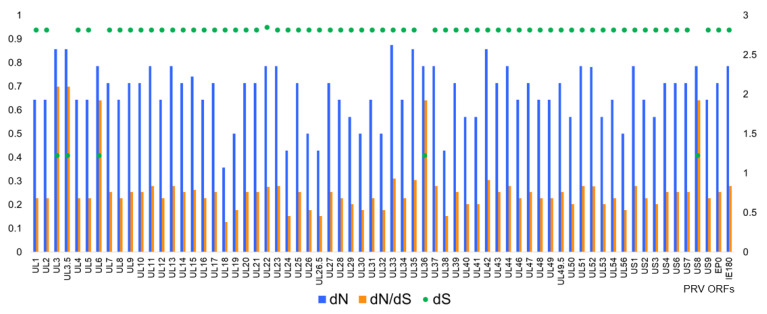
Selection pressure analysis. Selection pressure of each ORF of PRV was analyzed. ORF sequences were extracted from each annotated genome. By using FUBAR in the Datamonkey web server, nonsynonymous substitutions per nonsynonymous site (dN), synonymous substitutions per synonymous site (dS), and the dN/dS ratio of each ORF were calculated. The result is plotted as a bar graph. The dN and dN/dS ratio read the major *y*-axis at the left side, and dS takes the minor *y*-axis at the right side.

Intermolecular recombination is an important mechanism to increase genomic diversity, while spontaneous mutation caused by polymerase infidelity is another important mechanism leading to genome diversity [58]. A spontaneous mutation is accumulated by positive selection pressure but erased by negative selection pressure, which is also called purifying selection [58]. The ability to maintain the lifespan of persistent infection and its constant interaction with the host immune system highlights the substantial selective pressure on genome diversity. To characterize the overall selection pressure on each ORF, we determined the nonsynonymous substitutions per nonsynonymous site (dN), synonymous substitutions per synonymous site (dS), and the dN/dS ratio as evidence of purifying, neutral, or positive selection (Figure 5).

As shown in Figure 5, most PRV genes show evolutionary constraints. The average dN/dS ratio is 0.26. Only 7.5% of the 67 ORFs have a dN/dS ratio higher than 0.6, and 88% of the ORFs have a ratio less than 0.3. A recent study of herpes simplex virus 1 (HSV-1) also showed a predominance of the evolutionary constraint with an average dN/dS ratio at 0.27 [38], and similar results were found in beta- and gamma-herpesviruses [56,68]. In general, a low dN/dS ratio, representing evolutionary constraint or negative selection pressure, suggests the excellent adaptation of the parasite within their hosts, with mutations mostly leading to negative fitness effects and being removed effectively [56,58]. The evolutionary constraint possibly correlates with the low diversity of newly sequenced PRV genomes in this study. 

Five proteins exhibit a remarkably higher dN/dS ratio than that of most proteins: UL3, UL3.5, UL6, UL36, and US8 (Figure 5). The functions of UL3 and UL3.5 are largely unknown. In PRV, US8 (gE) shows close to neural selection or drift (dN/dS = 0.64), while the dN/dS ratio of US8 of HSV-1 is substantially lower (dN/dS = 0.19) [38]. gE is an essential neural virulence factor of PRV and one of the major targets of neutralizing antibodies. A relatively higher dN/dS ratio and diversity on gE imply that gE mutations may lead to antigenic drift or virulence enhancement. Considering all the PRV vaccine strains applied in pig farms have gE deletion, a higher dN/dS ratio in gE may result from the host immune response against wild PRV strains but not vaccination. In addition, nine ORFs (UL18, UL24, UL26.5, UL38, UL19, UL26, UL30, UL32, UL56) exhibit a relatively lower dN/dS ratio (lower than 0.2) (Figure 5). As expected, most of them are key components of DNA replication complex or essential virion structure proteins. 

In Table 3, we list the amino acid residues under positive selection determined by FUBAR, MEME, and CodeML of PAML. Interestingly, most ORFs bearing positive selection residues are essential for viral replication or virion assembly, while only UL23, US8, and EP0 are dispensable for viral replication in vitro [4]. In UL27 (gB), the Residues 75 and 505 show significant positive selection. Our alignment of UL27 shows that Residue 505 is proline in most strains, while four strains (Bartha-K61, Kolchis, Hercules, ADV32751) show alanine at this position (Figure 4C). A previous study demonstrated that region aa495–505 (PAAARRARRSP) is the furin cleavage site of gB [69]. The furin cleavage site of gB is not required for PRV viral replication in vitro but plays an essential role for the virus to spread between cells [69]. In human cytomegalovirus (HCMV), the positive selection of the furin cleavage site of gB leads to different furin cleavage efficacy [70]. However, the functional significance of positive selection at the furin cleavage site of gB in PRV is still unclear.

The residues under positive selection of UL27 (gB), UL44 (gC), and US8 (gE) were previously determined [36]. In their study, four positive selection residues of gB were detected by FUBAR and MEME (aa43, aa75, aa848, aa922), of which aa75 was also detected in our study [36]. Additionally, two positive selection residues were detected in gE (aa348, aa578). In our study, aa578 was also determined as positive selection sites in US8. No positive selection residues were detected in UL44 in our study, while two residues (aa59, aa194) were determined under positive selection in He’s study [36]. 

It should be noted that Residue 283 of UL9 is not only under positive selection (Table 3) but also a distinguishing mutation (A283T) (Table 2). This concordance strongly suggests that Residue 283 of UL9 might play a critical role in viral virulence enhancement.

## 4. Conclusions

In the study, we analyzed PRV genomic diversity and evolution with 73 PRV genomes, of which 54 genomes were newly sequenced in this study. Phylogenetic analysis divided PRV strains into two genotypes, which strongly supports geographical clustering and suggests PRV strains evolve independently in Asia and Europe. However, recombination analysis indicates that recombination between different genetic clades is robust, and the classic vaccine strain Bartha-K61 might contribute to the evolution process of novel PRV variants. Several distinguishing mutations were identified in 19 ORFs, which provided potential targets for further phenotype–genotype studies. Selection pressure analysis revealed that most ORFs of PRV are under strong purifying selection. Additionally, 19 amino acid residues were determined under positive selection within 15 different ORFs.

## Figures and Tables

**Table 1:** Nucleotide and amino acid diversity of PRV strains in open reading frames.

				Nucleotide				Amino Acid		
ORF	Common Name	Core Gene	Mean Distance (%)	Standard Error	Variable Sites	Length * (Nucleotide)	Ratio of Variable Sites	Mean Distance (%)	Standard Error	Ratio of Variable Sites
UL1	gL	Yes	2.66%	0.006	22	468	4.70%	3.10%	0.011	5.13%
UL2	UNG	Yes	0.72%	0.002	47	969	4.85%	0.89%	0.002	6.81%
UL3		No	2.00%	0.002	79	720	10.97%	3.79%	0.007	17.50%
UL3.5		No	1.64%	0.003	45	666	6.76%	2.80%	0.006	13.06%
UL4		No	0.84%	0.002	18	435	4.14%	1.27%	0.004	6.21%
UL5		Yes	0.23%	0.000	43	2505	1.72%	0.24%	0.001	2.16%
UL6		Yes	0.42%	0.001	44	1935	2.27%	0.72%	0.002	3.26%
UL7		Yes	1.13%	0.002	24	798	3.01%	0.19%	0.005	5.26%
UL8		Yes	1.60%	0.001	114	2064	5.52%	1.63%	0.003	7.70%
UL9	OBP	No	0.53%	0.001	104	2532	4.11%	0.83%	0.001	6.40%
UL10	gM	Yes	0.84%	0.002	34	1179	2.88%	1.80%	0.004	6.11%
UL11		Yes	1.67%	0.006	10	189	5.29%	2.94%	0.014	9.52%
UL12	AN	Yes	1.32%	0.002	79	1449	5.45%	2.15%	0.004	8.90%
UL13	PK	Yes	1.28%	0.002	51	1173	4.35%	2.29%	0.004	7.42%
UL14		Yes	1.30%	0.003	16	477	3.35%	2.20%	0.008	5.66%
UL15		Yes	1.38%	0.002	119	2226	5.35%	1.90%	0.002	7.68%
UL16		Yes	2.00%	0.003	48	984	4.88%	2.80%	0.006	7.01%
UL17		Yes	1.25%	0.002	83	1800	4.61%	1.82%	0.003	6.67%
UL18	VP23	Yes	0.76%	0.002	24	888	2.70%	0.58%	0.003	2.36%
UL19	VP5	Yes	0.45%	0.001	84	3990	2.11%	0.47%	0.001	2.56%
UL20		No	1.90%	0.005	20	495	4.04%	3.10%	0.010	6.06%
UL21		Yes	1.48%	0.002	101	1599	6.32%	2.20%	0.004	9.57%
UL22	gH	Yes	0.22%	0.000	44	2064	2.13%	0.44%	0.001	4.22%
UL23	TK		0.49%	0.001	21	960	2.19%	0.83%	0.003	3.44%
UL24		Yes	0.83%	0.003	9	513	1.75%	0.84%	0.005	1.75%
UL25		Yes	0.72%	0.001	54	1608	3.36%	1.00%	0.002	4.85%
UL26	VP24	Yes	0.77%	0.001	62	1599	3.88%	0.92%	0.002	5.63%
UL26.5	VP22	Yes	1.19%	0.002	40	861	4.65%	1.20%	0.004	5.92%
UL27	gB	Yes	0.96%	0.001	105	2742	3.83%	1.67%	0.003	6.67%
UL28	ICP18.5	Yes	0.71%	0.001	66	2166	3.05%	0.98%	0.002	4.99%
UL29	ICP8	Yes	0.51%	0.001	87	3537	2.46%	0.56%	0.001	3.14%
UL30		Yes	0.47%	0.001	52	3144	1.65%	0.48%	0.001	2.10%
UL31		Yes	0.96%	0.002	16	813	1.97%	1.44%	0.005	2.95%
UL32		Yes	0.80%	0.002	33	1413	2.34%	0.88%	0.003	2.76%
UL33		Yes	1.30%	0.003	11	351	3.13%	2.23%	0.010	5.98%
UL34		Yes	2.06%	0.004	45	783	5.75%	3.20%	0.008	8.81%
UL35	VP26	Yes	1.45%	0.004	10	309	3.24%	3.60%	0.013	7.77%
UL36	VP1/2	Yes	1.87%	0.001	993	9489	10.46%	2.70%	0.001	14.64%
UL37		Yes	0.70%	0.001	100	2757	3.63%	1.32%	0.002	6.96%
UL38	VP19c	Yes	0.93%	0.002	32	1104	2.90%	0.77%	0.002	3.26%
UL39	RR1	Yes	0.82%	0.001	81	2364	3.43%	1.18%	0.002	5.58%
UL40	RR2	No	0.82%	0.001	25	909	2.75%	0.90%	0.003	3.30%
UL41	VHS	No	1.02%	0.002	34	1095	3.11%	1.08%	0.003	4.11%
UL42		Yes	0.81%	0.001	52	1155	4.50%	1.69%	0.004	9.09%
UL43		No	0.93%	0.001	45	1119	4.02%	1.46%	0.004	5.63%
UL44	gC	No	2.38%	0.003	108	1461	7.39%	4.20%	0.005	13.35%
UL46	VP11/12	No	1.63%	0.002	125	2085	6.00%	2.63%	0.003	9.64%
UL47	VP13/14	No	1.89%	0.002	150	2217	6.77%	2.60%	0.003	9.47%
UL48	VP16	No	0.97%	0.002	41	1239	3.31%	1.52%	0.004	5.33%
UL49	VP22	No	1.37%	0.003	40	729	5.49%	2.06%	0.005	8.23%
UL49.5	gN	Yes	4.10%	0.008	25	297	8.42%	7.56%	0.022	15.15%
UL50	dUTPase	Yes	1.40%	0.003	37	807	4.58%	2.10%	0.006	5.95%
UL51		Yes	2.06%	0.004	40	729	5.49%	4.38%	0.009	11.11%
UL52		Yes	1.81%	0.001	329	2910	11.31%	2.56%	0.002	14.74%
UL53	gK	No	1.60%	0.003	39	936	4.17%	2.38%	0.006	6.09%
UL54	ICP27	Yes	2.00%	0.003	73	1083	6.74%	2.80%	0.005	11.08%
UL56		No	2.87%	0.004	47	621	7.57%	2.93%	0.008	8.70%
US1	ICP22	No	2.49%	0.003	235	1332	17.64%	3.34%	0.005	24.55%
US2	28K	No	2.30%	0.004	40	768	5.21%	3.50%	0.009	8.59%
US3		No	0.83%	0.002	28	1002	2.79%	1.33%	0.004	4.19%
US4	gG	No	1.29%	0.001	142	1497	9.49%	1.70%	0.003	12.63%
US6	gD	No	0.83%	0.001	44	1206	3.65%	1.50%	0.003	6.72%
US7	gI	No	1.98%	0.002	99	1098	9.02%	3.00%	0.005	13.11%
US8	gE	No	1.78%	0.001	233	1734	13.44%	2.68%	0.004	16.26%
US9	11K	No	1.97%	0.005	12	294	4.08%	2.07%	0.011	4.08%
EP0	ICP0	No	0.79%	0.001	44	1104	3.99%	1.19%	0.003	5.98%
IE180	ICP4	No	0.64%	0.001	214	4425	4.84%	0.98%	0.001	6.98%

*: The ORF nucleotide sequence lengths of each ORF were collected from the reference genome of PRV Ea strain.

**Table 2:** Distinguishing mutations in newly sequenced PRV strains.

ORF Name	Mutation Position ^$^	Function
UL2	V107A *	UNG, DNA repair; Uracil-DNA glycosylase
UL3.5	**T173A ^&^**, A178T,	Viral egress (secondary envelopment); membrane-associated protein
UL5	H250R	DNA replication; UL5 is helicase subunit of UL5/UL8/UL52 helicase/primase complex;
UL9	A283T, W500R, P696T,	OBP, sequence-specific ori-binding protein, ATP-dependent helicase motif
UL13	**T169A**	VP18.8, protein-serine/threonine kinase
UL14	G145E	Virion tegument protein,
UL15	**D163N**	Interacts with UL33, UL28 & UL6; DNA viral concatemeric DNA cleavage/encapsidation; terminase subunit of the UL15/UL28 complex;
UL27	**T82A**, R451K, H560Q, T737A, V895A	Viral entry (fusion); cell–cell spread; glycoprotein B; type I membrane protein
UL36	**P/S292A, P/S/Q293A, R2286Q, A/G2068R**, A3059T/G	Viral egress (capsid tegumentation); major tegument scaffold; interacts with UL37 and capsid
UL37	M782V	Viral egress (capsid tegumentation); interacts with UL36
UL42	**T270A**	DNA replication; polymerase accessory subunit of UL30/UL42 holoenzyme
UL44	**G194E**	Glycoprotein C (gC), viral entry (virion attachment); type I membrane protein; binds to heparan sulfate
UL47	G67E, **A219T**	VP13/14, viral egress (secondary envelopment); tegument protein
UL49.5	**A49T**	Glycoprotein N (gN), immune evasion (TAP inhibitor); type I membrane protein; complexed with gM
US1	E211G, E260G	ICP22, acts as a regulator of gene expression
US4	**S82P**	Glycoprotein G (secreted)
US6	V338A,	Glycoprotein D (gD), viral entry (cellular receptor-binding protein); type I membrane protein
US8	G54D, P403A S517P	Glycoprotein E (gE), cell–cell spread; glycoprotein E; type I membrane protein; complexed with gI; C-terminus interacts with UL49; protein sorting in axons
IE180	ΔΔ349-350RG, A841P, A842S,	ICP4, gene regulation (transcription activator); immediate-early protein

*: The mutation sites listed in the table indicate that certain amino acid residues of every PRV strain isolated after 2011 except HuB1/CHN2017 are collectively different from the classic Chinese PRV strains Ea/Fa and SC, which distinguish the novel PRV variants from the classic Chinese PRV strains (Ea, Fa, and SC). ^&^: The mutation sites with bold letters indicate that certain amino acid residues of every PRV strain isolated after 2011 except HuB1/CHN2017 are collectively different from the classic Chinese PRV strains Ea, Fa, and SC and all EU/US strains. ^$^: Each amino acid residue number is the codon number in the corresponding ORF of reference genome Ea.

**Table 3:** Position of each codon under positive selection.

ORF Name	Codon Position ^$^	Detection Method
UL5	574	F*, M^&^
UL6	462, 464	F, C^#^
UL9	283	F, C
UL12	474	F, M
UL15	674	F, M
UL19	502	F, M
UL23	293	M, C
UL25	472	F, C
UL27	75	F, M, C
	505	F, C
UL36	561	F, M
UL39	4	F, M, C
UL52	662	F, C
	663	M, C
US8	575	F, C
	578	M, C
EP0	212	F, M
IE180	1462	F, M

F*: FUBAR, posterior probability >90%; M^&^: MEME, *p* < 0.1; C^#^: CodeML, *p* < 0.05. ^$^: All amino acid residue positions are the codon number in the corresponding ORF of reference genome Ea.

## Data Availability

The data presented in this study are available in article and supplementary material.

## Supplementary Materials

The following are available online at https://www.mdpi.com/article/10.3390/v13071322/s1, Table S1. PRV strains sequenced in this study. Table S2. Previously published PRV strains used in this study. Table S3. RDP results summary. Table S4. Recombination events detected in multiple PRV strains. Table S5. *P* value of each recombination event detected by RDP. Table S6. Location of gapped regions in each newly sequenced PRV genome.
