# Supplementary material for: Whole-Genome Sequence Analysis of Pseudorabies Virus Clinical Isolates from Pigs in China between 2012 and 2017 in China
Source: Viruses. 2021 Jul 8;13(7):1322. doi: 10.3390/v13071322 (PMC8310123; doi:10.3390/v13071322)
Supplement: Supplementary file 1 [file viruses-13-01322-s001.zip › Supplementary materials-viruses-1286962-final.pdf]

Supplementary Material

Table S1. Sequencing result summary

Strain name	GenBank accession	Country (province, city)	Clean reads*	Coverage >=100X[&]	Average read depth[$]
AnH1/CHN2015	MK618718	China (Anhui, Bozhou)	999951	99.50%	702.53
BJ1/CHN2014	MK642564	China (Beijing)	1010029	99.42%	715.26
FJ1/CHN2016	MK642565	China (Fujian, Fuqing)	3634132	99.86%	2844.84
GD1/CHN2016	MK642566	China (Guangdong, Yangchun)	1000030	99.37%	712.56
GD2/CHN2016	MK642567	China (Guangdong)	7381508	99.96%	4903.4
GD3/CHN2016	MK642568	China (Guangdong, Fuchun)	7239364	99.95%	5395.4
GD4/CHN2016	MK642569	China (Guangdong, Yangchun)	5117776	99.78%	3329.85
GX1/CHN2014	MK642585	China (Guangxi, Guigang)	6600062	99.94%	5533.9
HeN1/CHN2012	MK642583	China (Henan, Sanmenxia)	1000027	99.39%	673.32
HeN1/CHN2014	MK642584	China (Henan, Xinyang)	5540608	99.94%	5002.18
HeN2/CHN2014	MK642582	China (Henan, Xinyang)	1000001	99.36%	652.04
HeN3/CHN2014	MK787153	China (Henan, Luohe)	1009975	99.44%	703.94
HeN4/CHN2014	MK642581	China (Henan, Zhumadian)	5284104	99.88%	4563.02
HeN1/CHN2015	MK642580	China (Henan, Nanyang)	9043770	99.93%	7364.34
HeN2/CHN2015	MK642579	China (Henan)	6699664	99.95%	3760.48
HeN3/CHN2015	MK642578	China (Henan)	4875306	99.70%	1419.74
HeN1/CHN2016	MK642577	China (Henan, Zhenzhou)	6886382	99.94%	5169.51
HeN2/CHN2016	MK642576	China (Henan, Xinyang)	8670118	99.97%	6122.11
HeN3/CHN2016	MK642575	China (Henan, Xiping)	5209702	99.82%	3924.3
HuB1/CHN2015	MK642574	China (Hubei, Jinzhou)	6388198	99.93%	5722.73
HuB2/CHN2015	MK642573	China (Hubei, Wuhan)	4687502	99.98%	3776.56

HuB3/CHN2015	MK642572	China (Hubei, Wuhan)	5005256	99.93%	4436.24
HuB4/CHN2015	MK642571	China (Hubei, Wuhan)	4942368	99.95%	3178.75
HuB5/CHN2015	MK642570	China (Hubei, Wuhan)	5305164	99.87%	2822.75
HuB1/CHN2016	MK682662	China (Hubei, Wuhan)	6706254	99.84%	4929.4
HuB2/CHN2016	MK682663	China (Hubei, Wuxue)	5978404	99.88%	4646.5
HuB3/CHN2016	MK682664	China (Hubei, Yichang)	4443330	99.92%	3203.48
HuB4/CHN2016	MK682665	China (Hubei, Hanchuan)	3750212	99.89%	2350.76
HuB5/CHN2016	MK682666	China (Hubei, Zhongxiang)	5436836	99.99%	5144.62
HuB6/CHN2016	MK682667	China (Hubei, Anlu)	10246602	99.97%	7153.28
HuB7/CHN2016	MK682668	China (Hubei, Huangzhou)	8256864	99.95%	6910.98
HuB8/CHN2016	MK682669	China (Hubei, Qianjiang)	4817506	99.90%	3707.91
HuB1/CHN2017	MK682670	China (Hubei)	5919500	99.97%	5515.16
HuB2/CHN2017	MK682671	China (Hubei)	4720654	99.89%	3751.36
HuN1/CHN2015	MK682672	China (Hunan, Zhuzhou)	5142096	99.92%	3715.05
HuN1/CHN2016	MK682673	China (Hunan)	4527914	99.98%	4332.4
JS1/CHN2015	MK682674	China (Jiangsu, Huaian)	1000046	99.52%	686.17
JS2/CHN2015	MK682675	China (Jiangsu, Yanchen)	8014294	99.93%	5677.47
JS1/CHN2017	MK682676	China (Jiangsu)	5221760	99.94%	4628.69
JX1/CHN2014	MK787152	China (Jiangxi, Fuzhou)	4891750	99.91%	4012.34
JX1/CHN2015	MK787151	China (Jiangxi, Nanchang)	4875462	99.96%	3902.15
JX2/CHN2015	MK787150	China (Jiangxi, Fengcheng)	5157302	99.95%	4825.63
NMG1/CHN2014	MK787154	China (Inner-Mongolia, Chifeng)	4891176	99.96%	4416.46
SD1/CHN2015	MK787155	China (Shandong, Rizhao)	10897334	99.99%	8618.92

SD1/CHN2016	MK787156	China (Shandong, Rizhao)	4011868	99.89%	3021.05
SD2/CHN2016	MK787164	China (Shandong)	4042198	99.84%	2950.73
SHH1/CHN2015	MK787156	China (Shanghai)	1009951	99.28%	694.12
ZJ1/CHN2012	MK787158	China (Zhejiang, Quzhou)	1000045	98.94%	681.58
ZJ2/CHN2012	MK787159	China (Zhejiang, Lishui)	5774986	99.96%	4940.4
ZJ1/CHN2014	MK787160	China (Zhejiang, Lishui)	1010000	99.39%	629.41
ZJ2/CHN2014	MK787161	China (Zhejiang, Jinhua)	1000046	99.51%	467.61
ZJ1/CHN2015	MK787162	China (Zhejiang, Jiangshan)	6414140	99.97%	4910.84
ZJ1/CHN2016	MK787163	China (Zhejiang)	5229744	99.79%	3045.67
ZJ1/CHN2017	MK787149	China (Zhejiang, Jiangshan)	3243466	99.95%	1854.25

*: Clean reads were filtered reads. The raw reads contain the reads of host genome and irrelevant microorganisms. The raw reads were aligned with nucleotide database and all the reads from host genome or irrelevant microorganisms were recognized and removed. The reads left were called clean reads;

&: Coverage of Next-generation sequencing (NGS) describes the mean number of reads that align to, or "cover," known reference bases. The sequencing coverage level often determines whether variant discovery can be made with a certain degree of confidence at particular base positions. This column gives the percentage of the bases covered by more than 100 different reads, which indicates the confidence of genome sequencing.

$: Average read depth indicates how many reads, on average, are likely to be aligned at a given reference base position.

Table S2. Previously published PRV strains

Strain	GenBank accession	Isolation time	Isolation location	Reference
HN1201	KP722022.1	2012	China	[1]
HNB	KM189914.3	2012	China	[2]
HNX	KM189912.1	2012	China	[3]
HeN1	KP098534.1	2012	China	[4]
HLJ8	KT824771.1	2014	China	[5]
JS-2012	KP257591.1	2012	China	[4]
ZJ01	KM061380.1	2012	China	Direct submission
TJ	KJ789182.1	2012	China	[6]
Fa	KM189913.1	1990	China	[7]
Ea	KX423960.1	1990	China	[8]
SC	KT809429.1	1990	China	[5]
Kolchis	KT983811.1	2010	Greece	[9]
Hercules	KT983810.1	2010	Greece	[9]
PRV-MdBio	LT934125.1	2015	Serbia	Direct submission
ADV32751	KU198433.1	2014	Italy	Direct submission
NIA3	KU900059.1	1970s	Northern Ireland	[10]
Kaplan	KJ717942.1	1970s	United States	[11]
Becker	JF797219.1	1960s	United States	[12]
Bartha-K61	JF797217.1	1950s	Hungry	[12]

Table S3. RDP results summary

Recombination events	Break points	Major parent strain	Minor parent strain	Recombinant strain
Event 3	Start: 36919-36984<br>End: 38009-38069	HuB4/CHN2015	Bartha-K61	ZJ01
Event 4	Start: 127018-127204<br>End: 127585-127957	ZJ2/CHN2012	Bartha-K61	ZJ01
Event 6	Start: 59019-67134<br>End: 67803-68107	Fa	Bartha-K61	HeN1(KP098534)
Event 7	Start: 89345-90108<br>End: 90971-91404	SC	Bartha-K61	HeN1(KP098534)
Event 8	Start: 102899-104850<br>End: 105886-106341	HuN1/CHN2015	Bartha-K61	ZJ01
Event 10	Start: 120450-125939<br>End: 126971-127899	Ea	ZJ01	SD1/CHN2016
Event 11	Start: 114432-114529<br>End: 114747-115668	BJ1/CHN2014	Bartha-K61	ZJ01
Event 12	Start: 58184-58270<br>End: 58554-59737	Fa	Bartha-K61	JS-2012
Event 14	Start: 122860-125198<br>End: 125258-126722	SD1/CHN2015	Bartha-K61	ZJ01
Event 15	Start: 114238-114422<br>End: 114575-114751	Fa	Bartha-K61	JS-2012
Event 17	Start: 117979-120447<br>End: 125853-127068	Ea	Bartha-K61	13 strains*
Event 19	Start: 107098-107501<br>End: 107544-108287	Fa	Bartha-K61	HNX
Event 24	Start: 17578-34261<br>End: 36951-36984	ZJ01	GD2	ZJ01
Event 26	Start: 58773-59737<br>End: 59790-61127	ZJ01	Bartha-K61	JS-2012
Event 28	Start: 85057-99750<br>End: 99982-100629	Fa	Bartha-K61	HeN1(KP098534)
Event 30	Start: 90907-107069<br>End: 107135-107809	Fa	ZJ01	HeN2/CHN2015
Event 35	Start: 126436-126927<br>End: 126951-127891	Ea	ZJ01	HeN2/CHN2014
Event 36	Start: 17577-48341<br>End: 48959-77652	ZJ01	HuB1/CHN2017	JS-2012
Event 37	Start: 114662-116395<br>End: 117591-120490	HuB1/CHN2017	Bartha-K61	59 strains*
Event 40	Start: 75987-89029<br>End: 89060-103826	ZJ01	HuB1/CHN2017	HeN1(KP098534)

Event 41	Start: 34983-35237<br>End: 35270-35480	Fa	Bartha-K61	HeN1(KP098534)
Event 47	Start: 94202<br>End: 126883-127379	HNB	Bartha-K61	HuB1/CHN2017
Event 52	Start: 3016-17043<br>End: 17577-18367	ZJ01	HeN1/CHN2016	HuB1/CHN2017

*: The recombination strains were listed in Table S4.

Table S4. Recombination events detected in multiple PRV strains

Recombination event	PRV isolates with recombination events detected
Event 37	HeN1/CHN2014, HeN2/CHN2014, HeN3/CHN2014, HeN4/CHN2014, HeN5/CHN2014, HeN1/CHN2015, HeN2/CHN2015, HeN3/CHN2015, HeN1/CHN2016, HeN2/CHN2016, HeN3/CHN2016, ZJ1/CHN2012, ZJ2/CHN2012, ZJ1/CHN2014, ZJ2/CHN2014, ZJ1/CHN2015, ZJ1/CHN2016, ZJ1/CHN2017, HuB1/CHN2015, HuB2/CHN2015, HuB3/CHN2015, HuB4/CHN2015, HuB5/CHN2015, HuB1/CHN2016, HuB2/CHN2016, HuB3/CHN2016, HuB4/CHN2016, HuB5/CHN2016, HuB6/CHN2016, HuB7/CHN2016, HuB8/CHN2016, HuB2/CHN2017, JX1/CHN2014, JX1/CHN2015, JX2/CHN2015, GX1/CHN2014, BJ1/CHN2014, SHH1/CHN2015, JS1/CHN2015, JS2/CHN2015, JS1/CHN2017, GD1/CHN2016, GD2/CHN2016, GD3/CHN2016, GD4/CHN2016, SD1/CHN2015, SD1/CHN2016, SD2/CHN2016, AnH1/CHN2015, HuN1/CHN2015, HuN1/CHN2016, NMG1/CHN2014, FJ1/CHN2016, JS-2012, HNB, HNX, HLJ8, HeN1(KP098534), ZJ01
Event 17	HuB1/CHN2015, HuB5/CHN2016, HuB6/CHN2016, JS2/CHN2015, JS1/CHN2017, JX1/CHN2015, JX2/CHN2015, HeN1/CHN2016, NMG1/CHN2014, ZJ1/CHN2012, ZJ1/CHN2017, HNX, JS-2012

Table S5. *P* value of each recombination events detected by RDP

	Detection method						
	RDP	GENECONV	Bootscan	Maxchi	Chimaera	Siscan	3seq
Event 3	5.775×10^{-105}	8.064×10^{-112}	2.801×10^{-109}	1.163×10^{-20}	7.926×10^{-21}	4.903×10^{-20}	6.994×10^{-13}
Event 4	1.567×10^{-101}	4.913×10^{-112}	7.249×10^{-97}	5.779×10^{-19}	7.274×10^{-19}	2.376×10^{-17}	6.994×10^{-13}
Event 6	2.648×10^{-60}	2.979×10^{-59}	1.205×10^{-56}	6.170×10^{-16}	4.916×10^{-17}	5.989×10^{-10}	7.993×10^{-13}
Event 7	1.090×10^{-52}	2.889×10^{-53}	9.675×10^{-53}	4.163×10^{-11}	2.599×10^{-11}	1.308×10^{-10}	6.994×10^{-13}
Event 8	9.158×10^{-44}	6.572×10^{-30}	4.610×10^{-44}	6.512×10^{-11}	4.553×10^{-11}	NS	6.994×10^{-13}
Event 10	3.492×10^{-37}	3.510×10^{-39}	9.277×10^{-34}	2.828×10^{-6}	3.075×10^{-11}	8.329×10^{-5}	9.992×10^{-14}
Event 11	6.598×10^{-36}	7.524×10^{-39}	4.942×10^{-36}	1.785×10^{-6}	1.604×10^{-6}	6.140×10^{-4}	2.110×10^{-9}
Event 12	1.715×10^{-31}	2.156×10^{-33}	1.259×10^{-31}	5.001×10^{-9}	3.850×10^{-9}	7.644×10^{-7}	7.993×10^{-13}
Event 14	4.031×10^{-30}	9.180×10^{-27}	8.230×10^{-13}	1.242×10^{-8}	5.604×10^{-9}	8.816×10^{-15}	6.994×10^{-13}
Event 15	3.443×10^{-26}	3.384×10^{-29}	4.090×10^{-18}	5.720×10^{-5}	5.281×10^{-5}	9.344×10^{-5}	7.993×10^{-13}
Event 17	1.628×10^{-4}	1.770×10^{-24}	1.011×10^{-11}	2.984×10^{-6}	1.270×10^{-4}	7.208×10^{-28}	7.993×10^{-13}
Event 19	2.535×10^{-24}	2.019×10^{-22}	1.188×10^{-22}	8.218×10^{-4}	7.938×10^{-4}	NS	1.024×10^{-10}
Event 24	4.368×10^{-18}	2.538×10^{-12}	1.602×10^{-13}	4.005×10^{-7}	4.368×10^{-18}	4.368×10^{-18}	1.998×10^{-13}
Event 26	2.233×10^{-15}	4.994×10^{-14}	1.961×10^{-15}	4.807×10^{-2}*	4.277×10^{-2}	5.444×10^{-5}	1.929×10^{-6}
Event 28	2.213×10^{-15}	2.065×10^{-13}	2.127×10^{-15}	3.355×10^{-2}	1.385×10^{-2}	3.679×10^{-3}	1.241×10^{-7}
Event 30	4.811×10^{-14}	7.083×10^{-9}	7.089×10^{-13}	1.425×10^{-2}	3.205×10^{-3}	1.549×10^{-7}	4.869×10^{-7}
Event 35	2.208×10^{-12}	4.797×10^{-13}	2.120×10^{-12}	3.040×10^{-2}	1.641×10^{-2}	NS	2.967×10^{-5}
Event 36	1.296×10^{-8}	1.379×10^{-5}	7.290×10^{-11}	NS	NS	2.917×10^{-4}	5.648×10^{-3}
Event 37	2.784×10^{-4}	6.525×10^{-2}	6.165×10^{-5}	1.256×10^{-3}	9.087×10^{-4}	1.222×10^{-9}	1.883×10^{-4}
Event 40	2.067×10^{-7}	2.536×10^{-9}	1.624×10^{-7}	NS	5.054×10^{-3}	1.210×10^{-7}	4.627×10^{-4}
Event 41	NS	9.270×10^{-11}	5.338×10^{-11}	NS	NS	1.487×10^{-7}	5.091×10^{-4}
Event 47	2.325×10^{-4}	2.709×10^{-3}	5.363×10^{-4}	3.290×10^{-8}	2.669×10^{-13}	1.650×10^{-20}	1.155×10^{-3}
Event 52	3.921×10^{-6}	6.390×10^{-3}	1.246×10^{-6}	2.782×10^{-4}	1.004×10^{-3}	1.162×10^{-11}	4.756×10^{-4}

*: Positive threshold, $P<0.001$; All the P values larger than 0.001 were highlighted by gray.

Reference

1. Xiang, S.; Zhou, Z.; Hu, X.; Li, Y.; Zhang, C.; Wang, J.; Li, X.; Tan, F.; Tian, K., Complete Genome Sequence of a Variant Pseudorabies Virus Strain Isolated in Central China. *Genome Announcements* **2016,** 4, (2), e00149-16.

2. Yu, T.; Chen, F.; Ku, X.; Zhu, Y.; Ma, H.; Li, S.; He, Q., Complete Genome Sequence of Novel Pseudorabies Virus Strain HNB Isolated in China. *Genome Announc* **2016,** 4, (1), e01641-15.

3. Ye, S.; Shao, K.; Li, Z.; Guo, N.; Zuo, Y.; Li, Q.; Lu, Z.; Chen, L.; He, Q.; Han, H., Antiviral Activity of Graphene Oxide: How Sharp Edged Structure and Charge Matter. *ACS Appl Mater Interfaces* **2015,** 7, (38), 21571-9.

4. Ye, C.; Zhang, Q. Z.; Tian, Z. J.; Zheng, H.; Zhao, K.; Liu, F.; Guo, J. C.; Tong, W.; Jiang, C. G.; Wang, S. J.; Shi, M.; Chang, X. B.; Jiang, Y. F.; Peng, J. M.; Zhou, Y. J.; Tang, Y. D.; Sun, M. X.; Cai, X. H.; An, T. Q.; Tong, G. Z., Genomic characterization of emergent pseudorabies virus in China reveals marked sequence divergence: Evidence for the existence of two major genotypes. *Virology* **2015,** 483, 32-43.

5. Ye, C.; Guo, J. C.; Gao, J. C.; Wang, T. Y.; Zhao, K.; Chang, X. B.; Wang, Q.; Peng, J. M.; Tian, Z. J.; Cai, X. H.; Tong, G. Z.; An, T. Q., Genomic analyses reveal that partial sequence of an earlier pseudorabies virus in China is originated from a Bartha-vaccine-like strain. *Virology* **2016,** 491, 56-63.

6. Luo, Y.; Li, N.; Cong, X.; Wang, C. H.; Du, M.; Li, L.; Zhao, B.; Yuan, J.; Liu, D. D.; Li, S.; Li, Y.; Sun, Y.; Qiu, H. J., Pathogenicity and genomic characterization of a pseudorabies virus variant isolated from Bartha-K61-vaccinated swine population in China. *Vet Microbiol* **2014,** 174, (1-2), 107-15.

7. Yu, T.; Chen, F.; Ku, X.; Fan, J.; Zhu, Y.; Ma, H.; Li, S.; Wu, B.; He, Q., Growth characteristics and complete genomic sequence analysis of a novel pseudorabies virus in China. *Virus Genes* **2016,** 52, (4), 474-83.

8. Wang, X.; Wu, C.-X.; Song, X.-R.; Chen, H.-C.; Liu, Z.-F., Comparison of pseudorabies virus China reference strain with emerging variants reveals independent virus evolution within specific geographic regions. *Virology* **2017,** 506, 92-98.

9. Papageorgiou, K. V.; Suarez, N. M.; Wilkie, G. S.; Filioussis, G.; Papaioannou, N.; Nauwynck, H. J.; Davison, A. J.; Kritas, S. K., Genome Sequences of Two Pseudorabies Virus Strains Isolated in Greece. *Genome Announc* **2016,** 4, (1),

e01624-15.

10. Mathijs, E.; Vandenbussche, F.; Verpoest, S.; De Regge, N.; Van Borm, S., Complete Genome Sequence of Pseudorabies Virus Reference Strain NIA3 Using Single-Molecule Real-Time Sequencing. *Genome Announcements* **2016,** 4, (3), e00440-16.

11. Tombacz, D.; Sharon, D.; Olah, P.; Csabai, Z.; Snyder, M.; Boldogkoi, Z., Strain Kaplan of Pseudorabies Virus Genome Sequenced by PacBio Single-Molecule Real-Time Sequencing Technology. *Genome Announc* **2014,** 2, (4), e00628-14.

12. Szpara, M. L.; Tafuri, Y. R.; Parsons, L.; Shamim, S. R.; Verstrepen, K. J.; Legendre, M.; Enquist, L. W., A wide extent of inter-strain diversity in virulent and vaccine strains of alphaherpesviruses. *PLoS Pathog* **2011,** 7, (10), e1002282.
